# The societal cost and economic impact of surgical care on patients’ households in rural Uganda; a mixed method study

**DOI:** 10.1186/s12913-021-06579-x

**Published:** 2021-06-09

**Authors:** Obieze Nwanna–Nzewunwa, Rasheedat Oke, Esther Agwang, Mary-Margaret Ajiko, Christopher Yoon, Melissa Carvalho, Fred Kirya, Elliot Marseille, Rochelle A. Dicker

**Affiliations:** 1 Department of Surgery, Maine Medical Center, 22 Bramhall Street, Portland, ME 04102 USA; 2 Program for the Advancement of Surgical Equity, Department of Surgery, University of California Los Angeles, Los Angeles, CA 90095 USA; 3 Department of Surgery, Soroti Regional Referral Hospital, Soroti, Uganda; 4 David Geffen School of Medicine, University of California Los Angeles, Los Angeles, California USA; 5 Principal, Health Strategies International, Oakland, California USA

**Keywords:** Surgical care cost, Societal cost, Catastrophic medical expenditure, Uganda, Africa, Epidemiology, Global surgery

## Abstract

**Background:**

The epidemiology and cost of surgical care delivery in low-and middle-income countries (LMICs) is poorly understood. This study characterizes the cost of surgical care, rate of catastrophic medical expenditure and medical impoverishment, and impact of surgical hospitalization on patients’ households at Soroti Regional Referral Hospital (SRRH), Uganda.

**Methods:**

We prospectively collected demographic, clinical, and cost data from all surgical inpatients and caregivers at SRRH between February 2018 and January 2019. We conducted and thematically analyzed qualitative interviews to discern the impact of hospitalization on patients’ households. We employed the chi-square, t-test, ANOVA, and Bonferroni tests and built regression models to identify predictors of societal cost of surgical care. Out of pocket spending (OOPS) and catastrophic expenses were determined.

**Results:**

We encountered 546 patients, mostly male (62%) peasant farmers (42%), at a median age of 22 years; and 615 caregivers, typically married (87%), female (69%), at a median age of 35 years. Femur fractures (20.4%), soft tissue infections (12.3%), and non-femur fractures (11.9%) were commonest. The total societal cost of surgical care was USD 147,378 with femur fractures (USD 47,879), intestinal obstruction (USD 18,737) and non-femur fractures (USD 10,212) as the leading contributors. Procedures (40%) and supplies (12%) were the largest components of societal cost. About 29% of patients suffered catastrophic expenses and 31% were medically impoverished.

**Conclusion:**

Despite free care, surgical conditions cause catastrophic expenses and impoverishment in Uganda. Femur fracture is the most expensive surgical condition due to prolonged hospitalization associated with traction immobilization and lack of treatment modalities with shorter hospitalization.

**Supplementary Information:**

The online version contains supplementary material available at 10.1186/s12913-021-06579-x.

## Background

Access to surgical care is a key indicator of the strength of a health system [1, 2]. Approximately 71% of the world’s population, chiefly in low-and middle-income countries (LMICs), lack access to affordable, safe, and timely essential surgical care [3]. In 2015, the World Health Assembly passed a resolution (WHA68.15) recognizing emergency and essential surgical care as a component of Universal Health Care (UHC) [4]. If UHC is to be achieved, political prioritization and investments in surgical care delivery must be made in LMICs.

Essential surgical care delivery in LMICs is cost-effective [5–7]. Historically, surgical care delivery has been branded expensive and not scalable. This view has adversely limited the funding and prioritization of surgical care delivery systems. Recent data, however, estimate potential cost savings of about 12.3 trillion dollars between 2015 and 2030 will be realized if appropriate investments are made in surgical care delivery systems in LMICs [7].

To make appropriate investments in surgical health systems, stakeholders need a good understanding of the epidemiology and cost of surgical care delivery. Data on the economics and cost of surgical care delivery in LMICs is limited [7–10]. Such data would inform the prioritization of surgical care delivery within national health plans and assist the creation of National Surgical Obstetric and Anesthesia Plans (NSOAPs). Additionally, it would aid policy formulation, resource allocation to improve surgical access and quality in developing countries. Uganda, one of the poorest countries in the world, has a Gross Domestic Product (GDP) per capita of 642.78 United States Dollar (USD) and a population of about 43 million people [11, 12]. Despite free care at government hospitals, 80% of Ugandans are reportedly at risk of catastrophic or impoverishing medical expenditures from surgical care [13].

This study characterizes the cost of surgical care at the regional level from a societal perspective in rural Uganda. It aims to determine the societal cost of surgical care delivery and its drivers. “Societal cost” refers to all costs sustained by the society and comprises: patient and health provider costs, medical and non-medical costs, and productivity losses incurred to access surgical care [14, 15]. This study also seeks to ascertain the prevalence of catastrophic medical expenditures and medical impoverishment among surgical patients at Soroti Regional Referral Hospital (SRRH) and their households. Lastly, the study aims to elucidate the impact of surgical hospitalization on patients and their households.

## Methods

### Study setting

This prospective cohort study was conducted at SRRH, a 300-bed hospital regional hospital in the Teso subregion of Eastern Uganda which serves over 2 million people across its eight-district catchment area [2].

### Study subjects and enrolment

All surgical patients (excluding obstetric cases) admitted to SRRH between February 2018 and January 2019 and their caregivers were invited to participate in this study. Written informed consent was obtained from each study participant and/or caregiver. Prison inmates, persons who lacked the mental capacity to consent, or those who declined consent were excluded. For incapacitated patients or those under 18 years old, consent was obtained from their adult caregivers. All enrolled patients were followed from admission until discharge.

### Study instruments

*Instrument 1:* A data entry form to collect demographic (i.e. age, sex, occupation), clinical (diagnosis, complications, treatment received), investigations (lab tests and imaging) performed, and cost (transportation, out of pocket spending (OOPS)) data from all study participants.

*Instrument 2:* A data collection form to collect demographic and cost data from all patients’ caregivers during their hospitalization.

*Instrument 3:* A semi-structured qualitative interview guide comprising four open-ended questions to explore OOPS, the effect of the hospitalization on the patient’s family, occupation, and finances; and to determine sacrifices made by the patient or their household to access care.

### Data collection

Upon admission to the surgical ward, we collected demographic, clinical, and cost data from patients or their caregivers. We observed processes of care (medication administration, procedures performed, and resource utilization) and interviewed patients, caregivers, and providers to obtain data on treatments, complications, and expenses incurred daily. Data were collected on paper and then entered into Microsoft Excel [16].

Due to cultural sensitivity, the authors elected not to ask patients about their income but rather used prevalent household wages published by the Uganda National Household Survey (NHS survey) [17]. Other cost inputs used included water tariffs, medication costs, surgical procedure costs, administrative and ancillary costs, and personnel costs (detailed in Additional file 1 [17–21]). Cost data were obtained in Ugandan shillings (USh) and converted to USD using the prevalent World Bank 2018 exchange rate (USh 3727 per dollar) [21].

At discharge, we administered the qualitative survey to all patients or their caregivers. Interviews were conducted in English, Ateso, Swahili, or Luganda by a local researcher who was fluent in these languages. The same researcher translated non-English interviews into English.

### Data analysis

#### Quantitative data

Descriptive analyses were performed on demographic, clinical, and cost data and expressed as frequencies, medians, and proportions. Non-parametric data were analyzed with the Kruskal Wallis test. Proportions were analyzed with the Chi-square test and Fisher’s exact test. Finally, bivariate and multivariate linear regression models were built to identify predictors of the societal cost of surgical care.

The proportion of patients that incurred catastrophic medical expenditure (defined as OOPS > 40% household income) and suffered medical impoverishment (defined as households left with <$2.50 per day after OOPS) was estimated [22, 23]. Logistic regression models were built to predict catastrophic expenditures and medical impoverishment. All quantitative data were analyzed using Stata version 16 [24].

#### Qualitative data

Qualitative survey responses were transcribed on paper during the interview and subsequently transferred to Microsoft Excel for further analysis. Responses were analyzed using the grounded theory approach to identify key themes within each domain explored in the qualitative survey. The frequency of key responses was analyzed. OOPS determined via this interview were used to calculate catastrophic medical expenditures and medical impoverishment.

### Ethical considerations

This study was approved by the SRRH administration and ethics committee, and the Institutional Review Boards of the University of California, San Francisco, and the University of California Los Angeles.

## Results

In the 11-month study period, 548 patients were admitted to SRRH with a surgical condition; 546 (99.6%) of which were enrolled and followed until discharge. Two patients were excluded due to their inability to provide informed consent. All patient caregivers consented to be interviewed for this study.

### Demography of patients and caregivers

**Fig. 1:**
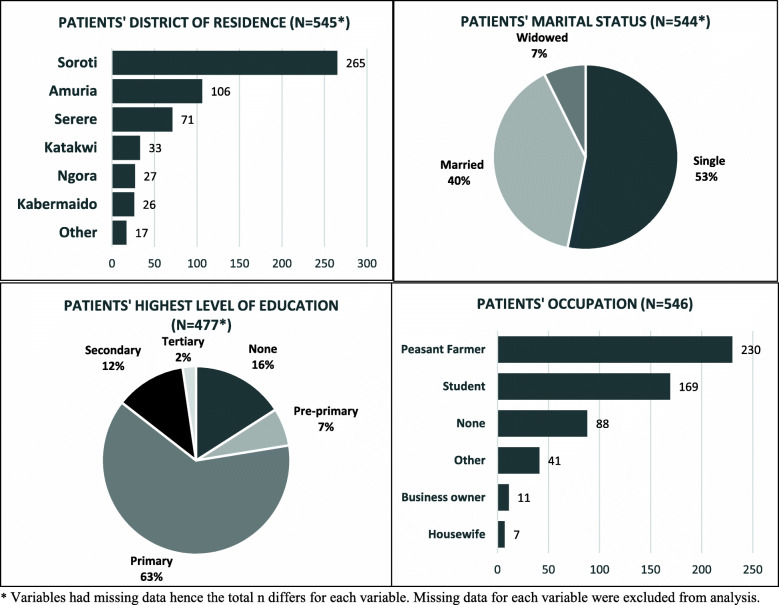
Demography of Surgical patients in SRRH

**Fig. 2:**
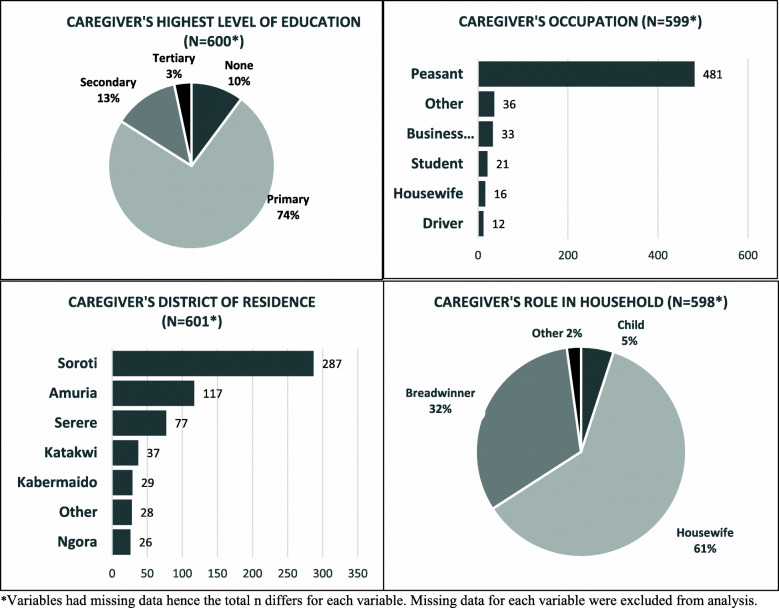
Demography of Surgical patients’ Caregivers

Over half, 62% (340/546) of the patients were males and 63% (301/477) had a primary level of education (Fig. 1). The patients had a median age of 22 years [IQR = 7, 49]. A total of 615 caregivers accompanied the 546 surgical patients, with an average of one caregiver (range: 0–5) per patient. Caregivers were typically married (87%, 523/601), female (69%, 414/601), at a median age of 35 years [IQR = 28, 45] with a primary level of education (74%, 443/600) (Fig. 2).

### Socioeconomic data

Patients were mostly peasant farmers (42%, 230/546) and students (31%, 169/546) who worked a median of 5 h [IQR = 4, 9] daily. Fifty-five percent (298/546) of patients reported having at least one dependent who relied on them financially for subsistence, with a median of 7 dependents [IQR = 4, 9]. Fifty-nine percent (313/546) of patients reported having at least one person who owned a mobile phone within their household. Caregivers were also mostly peasant farmers (80%, 481/599), businesspersons, or students (Fig. 2). Most caregivers (91%, 540/594) had at least one dependent, with a median number of 7 dependents [IQR = 4, 9].

### Clinical data

**Fig. 3:**
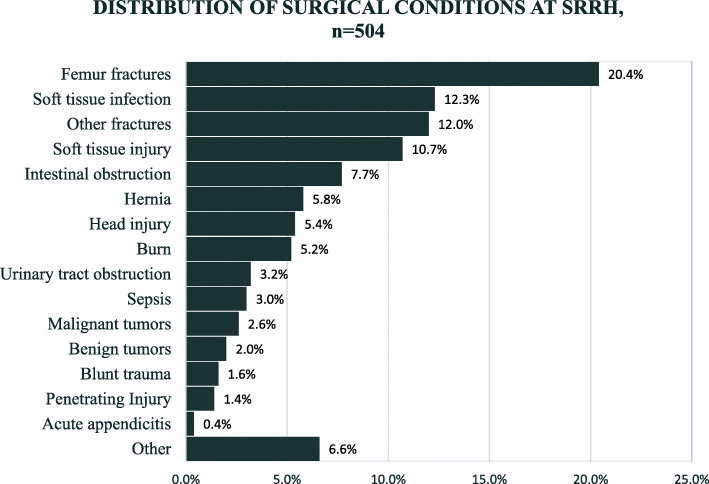
Prevalence of surgical conditions encountered at SRRH

Femur fractures (20.4%), were the most commonly encountered surgical condition among study subjects (*n* = 504) followed by soft tissue infections (12.3%), non-femur fractures (11.9%), soft tissue injuries (10.7%), intestinal obstructions (7.7%), and hernias (5.8%). Acute appendicitis was less common (0.4%) (Fig. 3). The median length of stay for all patients was 7 days [IQR =3, 17 days].

### Societal cost of surgical care at SRRH

The direct health costs captured include inpatient accommodation, surgery or procedure(s), medication(s), medical supplies, clinical staff, administrative and ancillary personnel. Indirect costs included lost productivity and transportation costs to and from the hospital.

**Fig. 4:**
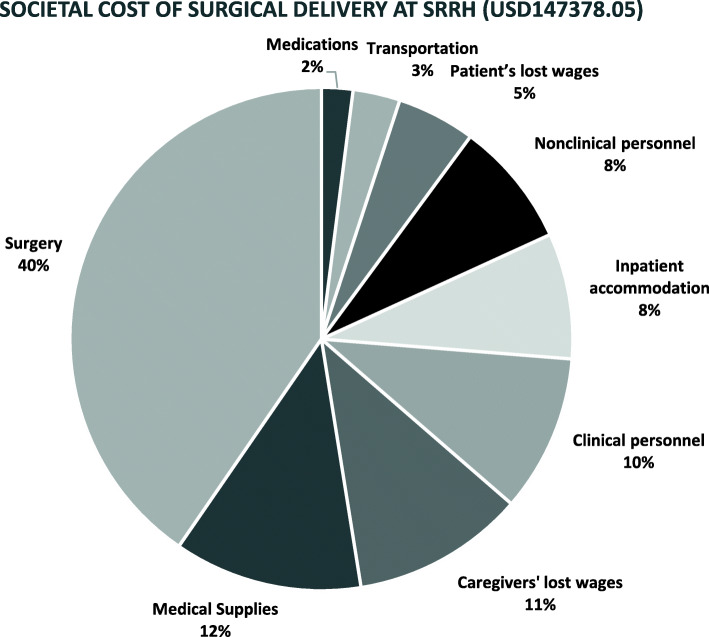
Composition of the societal cost of surgical care delivery at SRRH

The total societal cost of surgical care delivery to the 546 patients over the study period was USD 147,378. The largest proportion (40%) was attributable to surgical procedures (USD 58,951) followed by medical supplies (USD17,685, 12%), and the caregivers’ lost wages (USD 16,212, 11%). The total transportation cost was USD 4421 (3%), while medications (2%) and utilities (1%) were among the least expensive components of societal cost, amounting to USD 2949 and USD 1474 respectively (Fig. 4).

From the societal perspective, the highest proportion of total cost attributable to a single surgical condition (32.5%) was due to femur fractures (Table 1), which affected 103 patients and costed a total of USD 47,879. Next in line was intestinal obstruction (13%) which affected 39 patients with a total cost of USD 18,737, soft tissue infections (USD 14,249), and other (non-femur) fractures (USD 10,212). Burns (USD 260) and acute appendicitis (USD 337) were the least expensive conditions.

**Table 1:**
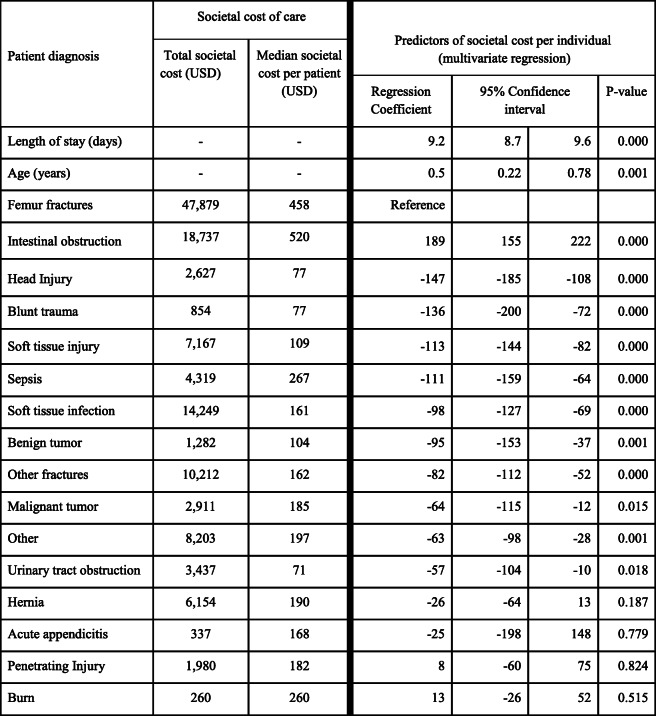
Total and median societal cost of surgical conditions and predictors of societal cost of surgical care delivery by diagnosis

### Predictors of societal cost of surgical care

Bivariate regressions analyses indicated length of hospitalization, diagnosis, and age to be significant predictors of societal cost. Multivariate analyses then revealed that length of hospitalization was the strongest positive predictor of societal cost. The societal cost of care increased USD 9.20 for each day of hospitalization and USD 0.5 for each additional year of age. Excluding intestinal obstruction, femur fractures cost significantly more than all surgical conditions (i.e. other fractures, sepsis, head injury, hernia, soft tissue injury, soft tissue infections, malignancies, benign tumors, and urinary tract obstruction) as demonstrated in the regression table (Table 1).

### OOPS, catastrophic expenditures and medical impoverishment

The median OOPS was USD 0 [IQR 0, 54], and about 38% of respondents (*n* = 385) reported some OOPS totaling USD 26,873. About 29% of respondents incurred catastrophic expenditures (i.e. expenditures > 40% of household income) while 31% suffered medical impoverishment. Education was protective against catastrophic medical expenditures (OR 0.99, *p* = 0.011) and medical impoverishment (OR 0.99, *p* = 0.04). Soft tissue injuries (OR 0.2, *p* = 0.001), soft tissue infections (OR 0.08, *p* < 0.0001), hernias (OR 0.1, *p* = 0.004), head injuries (OR 0.07, *p* = 0.014), burns (OR 0.19, p = 0.04), and non-femur fractures (OR 0.3, p = 0.004) all had lower odds of catastrophic expenditure than femur fractures. No surgical condition had a statistically significant higher likelihood of catastrophic expenditure than femur fractures.

### Qualitative interview

We interviewed all 546 patients or their designee to evaluate the impact of the surgical condition and hospitalization on the patient and their household. The themes elicited in the qualitative interviews fell under four domains (Table 2). Interviewees reported that their hospitalizations had adverse effects on their family and personal life such as adverse mental health effects (17%), family disruption/ dysfunction (14%), and death (2.6%). They also reported missing work (65%) and education (19%), income loss (57%), financial strain (23%), and depleted savings (16%). Only 2% of respondents reported having paid medical leave. A respondent said, “*We sold our land (USD 805) to access treatment”* (Respondent, LOS032). Another said, *“We sold food stuff (USD 54), 2 goats (USD 97), a bull (USD 258), a pig (USD 43)”* (Respondent, LOS031).

**Table 2:**
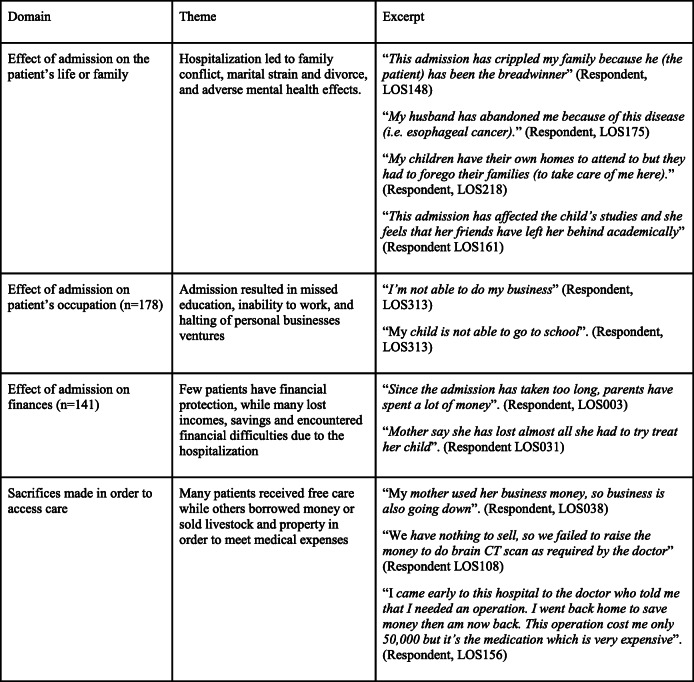
Themes elicited in the qualitative interview of surgical patients or their caregivers (*n* = 385)

## Discussion

Our findings indicate that the majority of surgical patients at SRRH are individuals in their productive years of life who get injured, have long hospitalization, and are thus out of the workforce. While most accessed free healthcare, a significant proportion of patients and caregivers incurred OOPS and lost wages that ultimately exceeded the intended financial protection offered by the Ugandan government through free public healthcare. Consequently, there is a significant rate of catastrophic expenses and medical impoverishment among surgical patients at SRRH.

A third of households incurred catastrophic medical expenditures and suffered impoverishment. The median societal cost of surgery was USD 201, which is five times the median income in the Teso subregion of Uganda; thus the absence of free care would impose significant financial hardship on surgical patients and their households [17]. A common reason for OOPS was the purchasing of medications outside the hospital during stockouts, indicating an opportunity for forecasting, supply chain, and service delivery improvement. Among our study subjects, increasing levels of education appeared to be the only factor that protected against catastrophic expenditures and medical impoverishment. We believe this is because a higher level of education is a proxy for higher socioeconomic status and income.

Cumulatively, femur fractures are the single most expensive surgical condition in the surgical health system of the Teso subregion of Eastern Uganda. Although the median per capita cost for intestinal obstruction (USD 520) was higher than that of femur fractures (USD 458), the total societal cost of femur fractures (USD 47,879) exceeded that of intestinal obstruction (USD 18,737) because femur fractures were three times more frequent than intestinal obstruction. Further, the length of hospitalization of femur fractures was about four times that of intestinal obstruction, resulting in significantly higher accommodation costs and lost wages to both patients and their caregivers. Despite being the commonest surgical condition encountered, the only treatment option available for femur fractures at SRRH is traction immobilization. Traction immobilization keeps patients bed-bound and has a longer hospitalization duration (30–52 days) and worse clinical outcomes (e.g. pneumonia, nonunion, malunion, etc.) than open fracture treatment methods like intramedullary nailing and open reduction with internal fixation (ORIF) with hospitalization duration of 3–6 days [2, 25–28]. Since the length of hospitalization is the strongest predictor of the cost of surgical care, it is important to explore alternative treatment options to decrease hospitalization duration, complications, cost, catastrophic expenditures, and medical impoverishment among surgical patients.

Prioritizing and investing in femur fracture management techniques with shorter hospitalization, rehabilitation, and better outcomes than femur traction would benefit patients and potentially the Ugandan economy. Agriculture accounts for about 69% of jobs and 26% of Uganda’s GDP [29]. About 50% of patients and 80% of attendants were farmers who could not work or contribute to the economy due to hospitalization. Techniques like intramedullary nailing and ORIF are associated with a shorter length of hospitalization and better outcomes than femur traction and may ultimately offer superior financial and clinical benefits to patients in rural Uganda, but will require investments from the government and stakeholders. Therefore, there is a need to evaluate the cost-effectiveness of intramedullary nailing or ORIF relative to femur traction in order to make an investment case for open fracture treatment.

Beyond the financial impact, prolonged hospitalization caused family conflict, emotional stress, and mental health challenges. Occasionally, it would cause family strain when a partner would be tied to the hospital bed in traction and preventing them from performing their family duties. This and other factors were a source of emotional stress. Unfortunately, social work and mental health support services are poorly developed in many LMIC settings.

### Limitations of the study

For sociocultural reasons, we did not inquire about patient income, but used the Ugandan national survey income data and adjusted the wages conservatively to reflect the occupation distribution of the participants and this may underestimate the income and lost wages due to hospitalization. Obstetric patients were housed in a separate building from the non-obstetric female surgical patients; for logistic reasons, obstetric patients were excluded. Thus, findings may not be generalizable to the obstetric population. Also, the qualitative interviews were transcribed during the interview session. While this may be an efficient way to handle a large sample of interviewees such as in our study, there is a risk of losing some information. However, this may be mitigated in part by the fact that vital and pervasive information would be a recurring theme in such a large sample.

## Conclusion

The cost of surgical care in rural Uganda is immense. Surgical patients at SRRH are vulnerable to, and suffer, medical impoverishment and catastrophic expenses. Without free public healthcare, such impoverishment would be commoner and worse. This study also demonstrates that femur fracture has the highest societal cost of surgical care and that the length of hospitalization is a key driver of the societal cost of surgical care delivery. There is thus a need to investigate the cost-effectiveness of open surgical treatment techniques relative to femur traction in this setting; the results of which could guide health care investments, policy formulation, and resource allocation.

## Supplementary Information

**Additional file 1: Appendix 1.** Sources of cost inputs used in cost analysis.

## Data Availability

The datasets used during the current study are available from the corresponding author on reasonable request.

## Abbreviations

GDP: Gross Domestic Product
IQR: Interquartile Range
LMICs: Low- and Middle-Income countries
NSOAPs: National Surgical Obstetric and Anesthesia Plans
OOPS: Out Of Pocket Spending
ORIF: Open Reduction with Internal Fixation
SRRH: Soroti Regional Referral Hospital
UHC: Universal Health Care
USh: Ugandan Shillings
USD: United States Dollar
