# Supplementary material for: The societal cost and economic impact of surgical care on patients’ households in rural Uganda; a mixed method study
Source: BMC Health Serv Res. 2021 Jun 9;21:568. doi: 10.1186/s12913-021-06579-x (PMC8190862; doi:10.1186/s12913-021-06579-x)
Supplement: Supplementary file 1 — Additional file 1: Appendix 1. Sources of cost inputs used in cost analysis. [file 12913_2021_6579_MOESM1_ESM.docx]

**Appendix 1: Sources of cost inputs used in cost analysis**

| **Cost input** | **Source** |
| --- | --- |
| Income/ local wages | Uganda National Household Survey^17^ |
| Hospital accommodation cost | World Health Organization. WHO-CHOICE estimates of cost for inpatient and outpatient health service delivery^18^. |
| Ancillary and administrative | Bellamkonda N, et al. Cost-Effectiveness of Exploratory Laparotomy in a Regional Referral Hospital in Eastern Uganda^19^ |
| Personnel cost | ﻿Bellamkonda N, et al. Cost-Effectiveness of Exploratory Laparotomy in a Regional Referral Hospital in Eastern Uganda^19^ |
| Water tariff | Uganda National Water and Sewerage Corporation^20^ |
| Cost of surgical procedure | Primary data from local providers |
| Exchange rate (USh to USD) | World Bank Database^21^ |
